# Treatment and Patient Reported Outcome in Children with Hirschsprung Disease and Concomitant Congenital Heart Disease

**DOI:** 10.1155/2017/1703483

**Published:** 2017-03-08

**Authors:** Johan Hasserius, Josefine Hedbys, Christina Graneli, Kristine Hagelsteen, Pernilla Stenström

**Affiliations:** ^1^Faculty of Medicine, Lund University, Lund, Sweden; ^2^Department of Clinical Sciences, Unit of Pediatric Surgery, Skane University Hospital, Lund University, 221 85 Lund, Sweden

## Abstract

*Purpose*. Congenital heart disease (CHD) is reported to be associated with Hirschsprung disease (HD). The aim was to evaluate any differences between children with HD with and without CHD, respectively, with regard to patient characteristics, medical care, and patient reported bowel function.* Method*. This is a retrospective chart study and a cross-sectional long-term follow-up of patients older than 4 years old, including all children with HD operated on with transanal endorectal pull-through (TERPT) at a tertiary center of pediatric surgery. Information about patient characteristics, diagnostics, surgery, and medical care was compiled. At long-term follow-up, bowel function was assessed by Bowel Function Score.* Results*. Included were 53 HD-patients, 13 with CHD and 40 without CHD. Children with CHD more commonly presented with failure to thrive; 4 (23%) compared to those without CHD (0%) (*p* < 0.01). In the long-term follow-up, including 32 patients (6 with CHD), constipation was more commonly reported by children with CHD 5 (83%) than by children without CHD 4 (27%) (*p* = 0.01). No differences were shown in the other parameters such as fecal control and incontinence.* Conclusion*. HD-patients with CHD more commonly presented with failure to thrive and more frequently reported constipation than HD-patients without CHD. The findings indicate that HD-patients with CHD might need special consideration in their initial care and long-term follow-up.

## 1. Introduction

Concomitant congenital anomalies might have implications for the outcome in children with Hirschsprung disease (HD) [1]. Congenital heart disease (CHD) has been reported to be present in 4–17% of children with HD [1–4]. There are no reports of whether CHD affects the medical care or surgical course in children with HD or if the outcome, with regard to bowel function, differs between patients with HD with and without CHD. Such knowledge could come to play an important role in optimizing the treatment and follow-up of children with HD and CHD.

The aim of this study was to investigate if there were any differences between HD-patients with and without CHD, respectively, operated on with transanal endorectal pull-through (TERPT), with regard to birth characteristics, initial symptomatology, diagnostic and surgical procedures, short-term postoperative outcome, and long-term outcome with regard to bowel function.

## 2. Method

### 2.1. Settings

The study was conducted at a tertiary pediatric surgical center, which covers 2 million residents and 25000 live births/year. The center is also a National Specialized Medical Care center for pediatric cardiology and cardiac surgery. The design of the study was dual: one part was a retrospective study of the children's records and the other part was a cross-sectional long-term follow-up comparing bowel symptoms in children with HD with or without CHD, respectively.

### 2.2. Patients

All children diagnosed with HD and operated on with TERPT from July 2005 until July 2015 were included in the study. TERPT was implemented as the main reconstruction procedure at the department of pediatric surgery in July 2005. Excluded were children operated on with methods other than TERPT, children with total colonic aganglionosis, and children who had been operated on at other centers, as well as those who had emigrated from the region.

### 2.3. Retrospective Study

Pre- and postoperative data were collected from the patient's records regarding initial symptoms, methods of diagnostics, preoperative evaluations, and waiting time for operation. Postoperative outcomes during 1 year after operation were registered. Complications, time at the hospital before discharge, and number and types of follow-up counseling were compiled from a prospectively collected database. Follow-up counseling was defined as planned outpatient clinic counseling or unplanned emergency counseling at the emergency department and, also, hospital stays. Reoperations and additional operations, such as appendicostomy, were registered for the whole period until long-term follow-up.

### 2.4. Long-Term Functional Follow-Up

In the long-term follow-up, children with stoma and children younger than 4 years of age were excluded. The children's bowel function was assessed by the Bowel Function Score (BFS) questionnaire. The questionnaire, which was evaluated and used in previous reports, consisted of 7 questions with 3 or 4 answer alternatives, which in the end lead to both a score on each question (0–3) and also a combined total BFS (1–20, 20 = best) [5–7]. The questionnaire was completed during regular counseling, either by telephone or at the outpatient clinic. A trainee who had not been involved in the care interviewed the HD-patients, and they answered the questions together with their parents.

### 2.5. Diagnostics and Operations

Rectal suction biopsies or full-thickness rectal biopsies were taken preoperatively and presence of hypertrophic nerve cells and aganglionosis as well as negative staining with calretinin [8, 9] was mandatory for diagnosis. Contrast enema was performed by using cold contrast in order to disclose the rectoanal relaxation reflex and to determine the level of the transition zone [10]. TERPT was performed via a transanal approach and some were performed with laparoscopic assistance. Transanal mucosectomy was performed starting approximately 10 mm above the dentate line and continuing for approximately 2–4 cm. A coloanal anastomosis was established with hand-sewn technique. Three pediatric surgeons, all with colorectal profiles, performed all the operations. These surgeons were also the ones who were responsible for preoperative evaluation, postoperative care, and clinical follow-ups.

Postoperative calibration of the anastomosis started 4 weeks after TERPT according to the local follow-up program. All children went into a bowel management program, where evaluation of bowel function was performed continuously during childhood. According to this program, the indication for appendicostomy was a desire for greater autonomy in patients regularly treated with colonic washouts or when patients in need of enemas experienced strong unwillingness to a rectal approach because of anatomical or psychological reasons.

### 2.6. Definitions

CHD was defined as any cardiac failure or cardiac anomaly diagnosed with cardiac ultrasonography by a cardiologist. Delay of meconium release was defined as if no meconium had passed spontaneously within 24 hours after birth. Enterocolitis was defined as fever (>38°), distended bowel, and foul-smelling stool requiring antibiotics and enema as treatment. Congenital syndromes were defined as named and diagnosed syndromes with more than 2 anomalies or chromosomal aberrations, while cognitive disability was defined as any learning problem and mental developmental delay reported by parents.

### 2.7. Statistics

IBM SPSS statistics, Version 23, Release 23.0.0.0, 64-bit edition, was used for statistical calculations. A statistician designed the study. Nonparametric Mann–Whitney* U* test was used for continuous data that was not normally distributed or when the sample size was small and for ordinal data. Fisher's exact test was used for binary categorical data. Median (range) was used for the description of continuous outcomes. A *p* value less than 0.05 was defined as statistically significant.

### 2.8. Ethics

The study was approved by the regional ethical review board (registration number 2010/49). All patients were evaluated and treated with standard care at the tertiary pediatric surgical center. All data was anonymized prior to calculation so that it would be impossible to identify any single patient. Approvals from the HD-patients' guardians and controls were obtained.

## 3. Results

### 3.1. Patients

**Figure 1:**
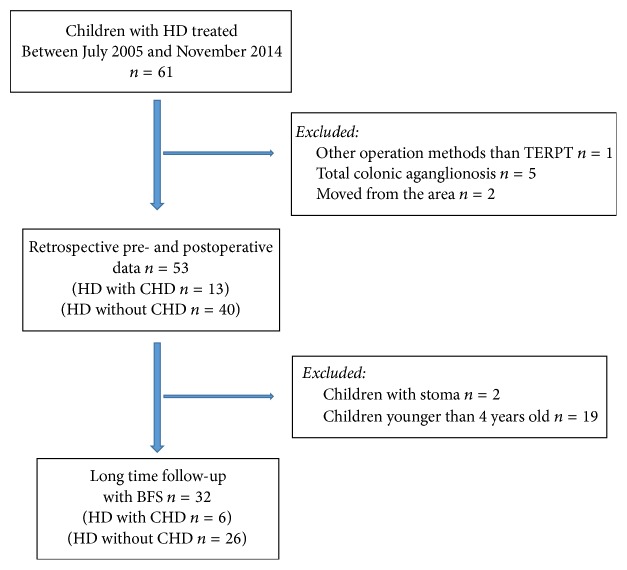
Flowchart for inclusion and exclusion of patients. TERPT: transanal endorectal pull-through, HD: Hirschsprung disease, and CHD: congenital heart disease.

Sixty-one patients were born with and operated on because of HD during the study period. 53 patients were included, of whom 13 (25%) had concomitant CHD (Figure 1). The different types of diagnosed CHD are listed (Table 1).

### 3.2. Birth Characteristics and Presenting Symptoms

**Figure 2:**
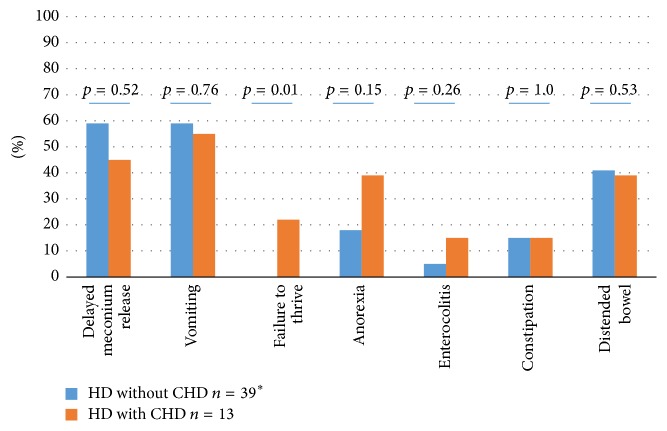
Presenting symptoms for 53 children with Hirschsprung disease (HD) with and without congenital heart disease (CHD). Number (%). Fisher's exact test. ^*∗*^Information missing in one of 40 children's records.

Regarding birth weight, gestation age, and gender, there was no statistical significant difference between HD-patients with and without CHD, respectively. The following syndromes and chromosomal abnormalities were registered: Down syndrome (*n* = 7), BRESCHEK (*n* = 1), chromosomal duplication (*n* = 1), chromosomal deletion (*n* = 1), epilepsia/hydrocephalus/mental retardation (*n* = 1), unspecified retardation, and delayed neuromuscular development (*n* = 1). The frequency of children with cognitive disability within a syndrome, which might be crucial for outcome, and the frequency of other concomitant malformations than CHD did not differ significantly between the groups (Table 2). Failure to thrive, as a presenting symptom, was more common among children with CHD (Figure 2).

### 3.3. Pre-and Postoperative Care and Surgical Approach

Age at first symptom of HD, age at first contact with a pediatric surgeon, and the time before the diagnose was set did not differ between patients with and without CHD. The age at surgery was 52 days for patients with CHD and 49 days for patients without CHD. Postoperative in-hospital time was median 4 days in both patient groups (Table 3).

Stoma before TERPT was established in 1 (8%) of the patients with CHD and in 8 (15%) without CHD (*p* = 0.66). A complete transanal approach was performed in 10 (77%) with CHD and in 35 (88%) without CHD (*p* = 0.39). A combined transanal approach and planned laparoscopy/laparotomy because of undefined transition zone or aganglionic bowel close to the left colonic flexure was performed in 3 (23%) with CHD and in 4 (10%) without CHD (*p* = 0.34). Change to additional laparoscopy/laparotomy during the operative course of transanal TERPT was performed in one child without CHD and in none with CHD.

### 3.4. Complications and Reoperations

The frequency of short-term complications after TERPT, such as perianal problems, did not differ between children with and without CHD. During the first year after TERPT, the frequency of planned or emergency counseling sessions, hospital stays, and need of anal dilatations did not differ between the two groups (Table 4). During the whole follow-up period, median 7 (4–10) years after surgery, none of the children with CHD needed any reoperation because of TERPT-complications but one child with CHD had severe nutritional problems and received a gastrostomy, and one had a neurogenic bladder, endocarditis, candida sepsis, aspiration pneumonia, and a laparotomy with stoma establishment due to toxic colitis. Five children (13%) without CHD needed reoperations because of complication due to TERPT: one child had a fistula between the vesicular seminales and rectum and received a colostomy that was closed before follow-up; one child had acute surgery due to leakage of the biopsy site on the bowel and received a diverting stoma that was closed before follow-up; one child had a twisted bowel postoperatively and was reoperated with a redo pull-through; one child with stricture developed a rectovesical fistula and therefore later received a permanent colostomy, still present at follow-up; one child with Down syndrome had a treatment resistant stricture, myectomy, and later received a permanent colostomy. Enterocolitis at any time was treated in 1 of the children with CHD and in 1 of the children without CHD (*p* = 0.43). The frequency of children with any complications overall, including all surgery, anal problems, enterocolitis, and other problems related to HD during follow-up, was among the children with CHD 9 (69%) compared to 28 (70%) among children without CHD (*p* = 1.00) (Table 4).

### 3.5. Long-Term Functional Follow-Up

In the long-term follow-up, bowel function was evaluated in 32 patients. CHD was present in 6 (19%). Median age was 8 (4–10) years in children with CHD and 6 (4–10) years in children without CHD. There were 2/6 (33%)* girls* with CHD and 7/26 (27%)* girls* without CHD (*p* = 1.00). Appendicostomy had been established in 2 (15%) of the children with CHD and in 5 (13%) of the children without CHD (*p* = 1.00). They were included in the long-term follow-up and were registered as in need of treatment with enemas. Constipation was significantly more commonly reported among children with CHD than among those without CHD. Children with CHD also more frequently reported daily soiling and impaired ability to hold back defecation, although significant differences were not reached (Table 5). When comparing items in BFS between children with HD and severe CHD (*n* = 3) (excluding those with mild CHD) with that of children with HD without any CHD disability to hold back defecation was reported weekly or absent by 3 (100%) and 12 (48%) (*p* = 0.03) and no constipation was reported by 0 (0%) and 19 (73%), respectively (*p* < 0.01). Further comparisons of BFS items between children with severe CDH and children without any CDH showed no statistical differences (urge to defecate *p* = 0.1, frequency of defecation *p* = 1.0, soiling *p* = 0.39, and fecal accidents *p* = 0.97). No children with HD and severe CHD answered the question of social problems.

## 4. Discussion

The results of the study show that more children with HD and concomitant CHD failed to thrive before HD-diagnosis was set, and they more frequently reported affected bowel function than HD-patients without CHD in long-term follow-up.

In the present study, 25% had a concomitant CHD. This prevalence of CHD is higher than that reported in previous studies on children with HD (4–17%) [1–4]. The higher frequency could be referred to the fact that our study was performed at a center for pediatric cardiac surgery and/or that the definition of CHD might be different compared to others. Further, the results showed that children with both HD and CHD more frequently presented with failure to thrive. Previous studies have shown that failure to thrive is a well-recognized problem for children with CHD without HD reporting on failure to thrive in 52%–61% of children with CHD [11–13]. This is more than in our study where 23% of the children with HD and CHD had failure to thrive. Similar, failure to thrive has been reported as one of the primary symptoms in children with HD who are diagnosed with HD later in life. In these children, failure to thrive has been reported in 24–62% at diagnosis but since there were no specific analyses for CHD, it remains unclear what role CHD had [14, 15].

Constipation as a problem in long-term follow-up after TERPT was reported inasmuch as 83% of the HD-patients with CHD compared to 27% without CHD. Even though constipation was more common among CHD patients, the constipation reported in both groups was mostly mild or laxative-controlled, rather than being severe. The rate of constipation has been reported to vary between 8 and 30% in children with HD operated on with TERPT but then without any analysis of frequencies of CHD in the cohorts [4, 5, 16, 17]. There are no reports on constipation rates in children with CHD, without HD, and therefore it is difficult to tell if this is a symptom of special concern in HD-patients. In our study a higher frequency of children with CHD reported daily soiling and inability to hold back defecation, and similar findings have not been published before. Overall, the total BFS, median of 11 and 14, respectively, was lower than in the previous studies where HD-patients reported a median BFS of 18 and 16, although this was without specifying the frequency of CHD [5, 18]. Possible explanations to why BFS overall was lower in our study could be that children of lower ages were included and that there was no dropout. Still, reasons for why children with CHD had even lower BFS could only be speculated. One reason could be that CHD might influence the hemodynamics in the bowel or that the medication for CHD might affect the bowel movements. The bowel function in children with CHD, without HD, has not been investigated and a controlled study would be of value.

Another interesting observation was that 100% of the children with HD and CHD reported no social problems because of HD, while 67% of the children with HD but without CHD did (although nonsignificant). In this study quality of life was not studied, but it could be expected to be lower in children who have both HD and CHD. However, previous studies have shown that the most important determining of quality of life in children and adolescents is development of coping strategies. Speculating, children with CHD and HD could have had strong reasons to develop coping strategies, to be able to deal with both cardiac and bowel symptoms. Those coping strategies might have resulted in their experience of a good social life. A previous study has shown that children with CHD self-reported better quality of life compared with the general population [19], although in a review article, quality of life was diversely reported by children with CHD compared to the general population [20]. Speculating, children with CHD might score differently from both children with HD and others because of being more affected by the CHD than other circumstances.

One aspect that could influence the outcome in HD is the cognitive disability. More children with CHD had a syndrome or chromosomal abnormality, but statistically the cognitive disability did not differ between the two groups. Not all children with syndromes or chromosomal abnormalities have cognitive disabilities, although in our study it is worth noting that all children with severe CHD also had a cognitive disability. This may of course have influenced their bowel control. Since the patients are few we cannot exclude that the difference in syndrome/chromosomal abnormalities also would influence the outcome in a larger study.

Lastly, the mortality among children with HD is almost eliminated in western countries, while mortality is still present among CHD patients. The combination of these abnormalities calls for urgent and larger studies including more controls with CHD, which could serve to increase knowledge in the field.

## 5. Conclusion

Children with HD and concomitant CHD more commonly present with failure to thrive and more frequently report affected bowel function than HD-patients without CHD. The findings indicate that children with HD and concomitant CHD need special consideration in initial care and long-term follow-up.

## Figures and Tables

**Table 1:** The different types of congenital heart diseases (CHD) identified among 13 out of 53 children with Hirschsprung disease (HD). PFO = patent foramen oval, ASD = atrial septal defect, and VSD = ventricular septal defect.

HD and CHD	Congenital heart disease
Patient 1	PFO or small ASD, with left to right shunt without restrictions^*∗*^
Patient 2	PFO with left to right shunt^*∗*^
Patient 3	Perimembranous ventricular septal defect (VSD) and ASD 4-5 mm
Patient 4	Muscular VSD, 3 mm
Patient 5	Dysplastic tricuspid valve with moderate insufficiency, pulmonic insufficiency, ductus with bidirectional flow^*∗∗*^
Patient 6	Small PFO and small ductus shunt^*∗*^; ^*∗∗*^
Patient 7	Small central ASD and atrial septal aneurysm^*∗∗*^
Patient 8	Bicuspid aortic valve^*∗∗*^
Patient 9	Aortic stenosis, dysplastic aortic valve stenosis
Patient 10	ASD 3-4 mm, minimal ductus^*∗*^; ^*∗∗*^
Patient 11	ASD without hemodynamic effect^*∗*^
Patient 12	Open PFO 4 mm, open ductus, left periphery pulmonary artery stenosis^*∗∗*^
Patient 13	ASD, obvious left to right shunt, 7 mm. Enlargement of both atriums and mild branch artery stenosis in arteria pulmonalis^*∗∗*^

^*∗*^Defined as mild forms of CHD.

^*∗∗*^Excluded in long-term follow-up because they were <4 years old.

**Table 2:** Summary of birth characteristics comparing children with Hirschsprung disease (HD) with and without congenital heart disease (CHD), presented as median (range) for birth weight and gestation age and as number (%) for gender, syndrome/chromosomal abnormality, cognitive disability within a syndrome, and congenital malformation. Pt = patients; *n* = number.

Birth data	HD without CHD *n* = 40		HD and CHD *n* = 13		*p* value
	*n*		*n*		
Birth weight (g)	36^*∗*^	3557 (2355–4675)	13	3344 (2100–4025)	*p* = 1.00^*∗∗∗*^
Gestation age (weeks)	34^*∗*^	39.5 (32–42)	12^*∗*^	39.0 (36–42)	*p* = 0.62^*∗∗∗*^
Gender (girls)	40	9 (23%)	13	3 (23%)	*p* = 1.00^*∗∗*^
Syndrome or chromosomal abnormality (pt)	40	3 (8%)	13	9 (69%)	*p* < 0.01^*∗∗*^
Cognitive disability (pt)	40	3 (8%)	13	3 (20%)	*p* = 0.34^*∗∗*^
Congenital malformations (pt)	40	3 (8%)	13	2 (15%)	*p* = 0.59^*∗∗*^
Number and types of malformations (*n*)		3 External ear malformation Congenital marmoreal telangiectasia cutis Morgagni hernia		5 Hearing deficit Vertebral anomaly Brain asymmetry Alopecia Ichtyosis	

^*∗*^Information was missing in some of the children's records.

^*∗∗*^Fisher's exact test.

^*∗∗∗*^Mann–Whitney *U* test.

**Table 3:** Summary of age and time intervals, counted in days, comparing the children with Hirschsprung disease (HD) with and without congenital heart disease (CHD) at different stages in the treatment process, presented as median number of days (range). TERPT = transanal endorectal pull-through.

	HD without CHD *n* = 40		HD and CHD *n* = 13		*p* value^*∗∗*^
	*n*		*n*		
Age at presenting symptom (days)	39^*∗*^	1.0 (0–228)	13	1.0 (0–167)	*p* = 0.37
Age at first contact with pediatric surgeon (days)	40	2.5 (1–1054)	13	5.0 (1–1119)	*p* = 0.37
Time from initial symptom to first counseling by pediatric surgeon (days)	38^*∗*^	2.0 (0–952)	13	4.0 (0–952)	*p* = 0.16
Time from birth to first counseling by pediatric surgeon (days)	40	3.0 (1–1054)	13	5.0 (1–1098)	*p* = 0.50
Age at first biopsy (days)	40	7.0 (1–1054)	13	10 (3–1138)	*p* = 0.46
Age at PAD result (days)	39^*∗*^	25 (8–1172)	13	21 (7–1159)	*p* = 0.50
Time between rectal biopsy and op-sign in (days)	37^*∗*^	5.0 (−2–367)	13	6.0 (0–90)	*p* = 0.46
Age TERPT (days)	40	48.5 (15–1254)	13	52 (12–1279)	*p* = 0.60
Time between first surgeon contact to operation reconstruction (days)	40	38.0 (12–888)	13	38.0 (9–160)	*p* = 0.48
Time from diagnosis, according to PAD, until TERPT (days)	39^*∗*^	27.0 (1–418)	13	18.0 (3–120)	*p* = 0.94
Days in hospital postoperatively (days)	40	4.0 (2–13)	13	4.0 (1–22)	*p* = 0.97
Time from discharge until first counselling (days)	40	11.0 (2–39)	13	10.0 (4–210)	*p* = 0.49

^*∗*^Information missing in children's records.

^*∗∗*^Mann–Whitney *U* test.

**Table 4:** Postoperative complications, number (%), and follow-up data, median (range), during the first year after transanal pull-through (TERPT) in children with Hirschsprung disease (HD) with and without congenital heart disease (CHD).

	HD without CHD *n* = 40		HD and CHD *n* = 13		*p* value
	*n*		*n*		
Anorectal complications (*n*)	40	24 (60%)	13	8 (62%)	*p* = 1.00^*∗∗*^
Skin excoriation (*n*)	40	22 (55%)	13	8 (62%)	*p* = 0.76^*∗∗*^
Stricture (*n*)	40	8 (22%)	13	2 (15%)	*p* = 1.00^*∗∗*^
Counseling sessions during the first 6 months after TERPT (*n*)	40	6 (1–30)	13	5 (2–9)	*p* = 0.14^*∗∗∗*^
Counseling sessions during the first year after TERPT (*n*)	40	8 (1–38)	12^*∗*^	7 (3–14)	*p* = 0.32^*∗∗∗*^
Planned counseling (*n*)	40	7 (1–26)	12^*∗*^	6 (2–11)	*p* = 0.11^*∗∗∗*^
Emergency counseling (*n*)	40	0 (0–6)	12^*∗*^	1 (0–2)	*p* = 0.28^*∗∗∗*^
Hospital stay (*n*)	40	1 (0–7)	12^*∗*^	1 (0–4)	*p* = 0.95^*∗∗∗*^
Calibrations first year (*n*)	40	6 (1–23)	12^*∗*^	6.5 (1–8)	*p* = 0.34^*∗∗∗*^
Dilatations first year (*n*)	40	0 (0–12)	12^*∗*^	0 (0–3)	*p* = 0.51^*∗∗∗*^

^*∗*^Patients missing because patient was operated on less than 1 year ago.

^*∗∗*^Fisher's exact test, two-tailed.

^*∗∗∗*^Mann–Whitney *U* test.

**Table 5:** Bowel function score (BFS) in children with Hirschsprung disease (HD) with and without congenital heart disease (CHD), median (range), aged 8 (4–10) and 6 (4–10) years, respectively, and the number of girls was 2 (33%) and 7 (27%), respectively. *N* = numbers and (%).

	Score	HD and CHD (*n* = 6)	HD without CHD (*n* = 26)	*p* value
*Ability to hold defecation*				
Always	3	0 (0%)	5 (19%)	*p* = 0.10^*∗∗∗*^
Problems <1/week	2	1 (17%)	9 (35%)	
Weekly problems	1	4 (67%)	11 (42%)	
No voluntary control	0	1 (17%)	1 (4%)	
*Feels/reports the urge to defecate*				
Always	3	2 (33%)	5 (19%)	*p* = 0.46^*∗∗∗*^
Most of the time	2	0 (0%)	13 (50%)	
Uncertain	1	3 (50%)	6 (23%)	
Absent	0	1 (17%)	2 (8%)	
*Frequency of defecation*				
Every other day to twice a day	2	2 (33%)	12 (46%)	*p* = 0.66^*∗∗*^
More often	1	3 (50%)	11 (42%)	
Less often	1	1 (17%)	3 (12%)	
*Soiling*				
Never	3	1 (17%)	0 (0%)	*p* = 0.08^*∗∗∗*^
Staining <1/week	2	0 (0%)	13 (50%)	
Frequent staining	1	2 (33%)	11 (42%)	
Daily/requires protective aids	0	3 (50%)	2 (8%)	
*Fecal accidents*				
Never	3	2 (33%)	13 (50%)	*p* = 0.33^*∗∗∗*^
Fewer <1/week	2	2 (33%)	7 (27%)	
Weekly	1	0 (0%)	5 (19%)	
Daily/requires protective aids	0	2 (33%)	1 (4%)	
*Constipation*				
No constipation	3	1 (17%)	19 (73%)	*p* = 0.01^*∗∗∗*^
Manageable with diet	2	2 (33%)	4 (15%)	
Manageable with laxatives	1	2 (33%)	2 (8%)	
Manageable with enemas	0	1 (17%)	1 (4%)	
*Social problems*		(*n* = 3)^*∗*^	(*n* = 24)^*∗*^	
No social problems	3	3 (100%)	16 (67%)	*p* = 0.24^*∗∗∗*^
Sometimes	2	0 (0%)	8 (33%)	
Restricting social life	1	0 (0%)	0 (0%)	
Severe social problems	0	0 (0%)	0 (0%)	
*Total BFS*		Median 11 range (11–15) (*n* = 3)^*∗*^	Median 14 range (11–20) (*n* = 24)^*∗*^	*p* = 0.15^*∗∗∗*^

^*∗*^2 and 3 patients in each group did not answer the question about social problems because of impaired cognitive disability which limited the answer rate in this question and also in total BFS.

^*∗∗*^Fisher's exact test.

^*∗∗∗*^Mann–Whitney *U* test.
